# A qualitative study of the process of adoption, implementation and enforcement of smoke-free policies in privately-owned affordable housing

**DOI:** 10.1186/s12889-019-7404-y

**Published:** 2019-08-08

**Authors:** Michelle C. Kegler, Erin Lebow-Skelley, Jaimie Lea, Regine Haardörfer, Adrienne Lefevre, Pam Diggs, Sally Herndon

**Affiliations:** 1 Department of Behavioral Sciences and Health Education, Emory Prevention Research Center, Rollins School of Public Health, Emory University, 1518 Clifton Road NE, Atlanta, GA 30033 USA; 2 Center for Maternal and Infant Health, School of Medicine, University of North Carolina at Chapel Hill, Chapel Hill, NC 27599 USA; 3 Director of Programs and Racial Equity, Youth Empowered Solutions [YES!], 4021 Carya Drive, Suite 160, Raleigh, NC 27610 USA; 4 North Carolina Department of Health and Human Services, Tobacco Prevention and Control Branch, Division of Public Health, 1932 Mail Service Center, Raleigh, NC 27699 USA

**Keywords:** Smoking, Policy, Tobacco, Affordable housing, Smoke-free policies, Qualitative

## Abstract

**Background:**

Household smoke-free home rules cannot fully protect nonsmokers from secondhand smoke (SHS) if they live in multi-unit housing (MUH). Instead, property-level smoke-free policies are needed to prevent SHS incursion into apartment units and to keep common areas smoke-free. Smoke-free policies are usually at the discretion of property management companies and owners within the context of market-rate and privately-owned affordable housing in the U.S.

**Methods:**

Semi-structured interviews on the policy development, implementation and enforcement experiences of 21 different privately-owned affordable housing management companies were conducted with representatives from properties in North Carolina and Georgia who had established smoke-free policies before 2016.

**Results:**

The decision to adopt was typically made by corporate leadership, board members, owners or property managers, with relatively little resident input. Policy details were influenced by property layout, perceptions of how best to facilitate compliance and enforcement, and cost of creating a designated smoking area. Policies were implemented through inclusion in leases, lease addenda or house rules with 6 months’ notice most common. Participants thought having a written policy, the norms and culture of the housing community, public norms for smoke-free environments, and resident awareness of the rules and their consequences, aided with compliance. Violations were identified through routine inspections of units and resident reporting. Resident denial and efforts to hide smoking were shared as challenges to enforcement, along with a perception that concrete evidence would be needed in eviction court and that simply the smell of SHS was insufficient evidence of violation. Over half had terminated leases or evicted residents due to violations of the smoke-free policy. The most common benefits cited were reduced turnover cost and time, and lower vacancy rates.

**Conclusions:**

Understanding the smoke-free policy process in privately-owned affordable housing can help practitioners encourage policies within subsidized housing contexts. The study identified salient benefits (e.g., reduced cost, time, and vacancies) that can be highlighted when encouraging MUH partners to adopt policies. Additionally, study findings provide guidance on what to consider when designing smoke-free policies (e.g., layout, costs), and provide insights into how to enhance compliance (e.g., resident awareness) and manage enforcement (e.g., routine inspections).

**Electronic supplementary material:**

The online version of this article (10.1186/s12889-019-7404-y) contains supplementary material, which is available to authorized users.

## Background

Residents of multi-unit housing (MUH) are often unable to fully protect themselves and their children from secondhand smoke (SHS) because SHS can travel between units through ductwork and shared hallways [1–4]. National data suggest that 34% of MUH residents who have their own smoke-free home rules have experienced smoke incursions into their units, with greater incursions reported by women, younger adults, African Americans, Hispanics and those with lower income [5]. Studies have also shown higher rates of incursion in government-subsidized units [6]. Since no amount of SHS is safe, public health efforts have focused on smoke-free policies in MUH as an effective approach to reducing SHS exposure [7–9]. In addition to health benefits, smoke-free policies in MUH offer cost savings, reduced turnover time, and reduced fire risk [10–12].

The U.S. Department of Housing and Urban Development (HUD) recently established a rule mandating smoke-free buildings and a 25-ft buffer zone in conventional public housing [13]. The rule will legally protect an estimated 2 million residents of conventional public housing [14]. The rule does not cover properties funded through mechanisms other than the Public Housing Program, such as the housing choice voucher program and the low income tax credit, meaning that a sizable proportion of residents of government-subsidized housing are not protected through the new rule. Moreover, a considerable proportion of this subsidized housing is privately-owned and often managed by property management companies. Privately-owned affordable housing, although not covered by the HUD rule, can voluntarily adopt smoke-free policies and momentum is building to do so [15–18].

Much of the research on smoke-free MUH has involved surveys of property managers, owners, and residents, to document levels of support and perceived barriers to such policies [10]. A few recent studies have evaluated the impact of newly established policies on SHS exposure and cessation rates, with mixed results and varying levels of rigor [12, 19–22]. Studies that carefully document the implementation and enforcement process across multiple settings are relatively rare [10, 16, 23]. In one of the larger studies of affordable housing, Stein and colleagues surveyed all affordable housing property companies in North Carolina and documented numerous aspects of both implementation and enforcement [15].

No qualitative studies have been conducted to understand the full policy adoption and implementation process across a diverse set of privately-owned affordable housing owners and management companies. A recent ethnographic study describes the compliance and enforcement process in four HUD-assisted properties managed by one private residential management company in Massachusetts [24], while another examined issues of compliance and enforcement within the context of two affordable housing environments in Canada, with an emphasis on the impact of lease exemption [23]. Given that a larger proportion of government-subsidized housing is privately-owned rather than managed through public housing authorities (PHA), understanding the policy development and implementation process in this context is valuable as efforts expand to support voluntary policies in the larger affordable housing arena. The current paper describes the policy development, implementation and enforcement experiences of 21 privately-owned affordable housing management companies with properties in North Carolina and Georgia, who had established smoke-free policies prior to the HUD rule.

## Methods

### Study participants

Our goal was to purposively sample at least 20 representatives from privately-owned affordable housing who had restricted smoking on at least one of their properties in North Carolina or Georgia. We also interviewed representatives from public housing authorities (PHAs) and those results are reported elsewhere [25, 26]. Eligible companies were identified through a combination of existing collaborations, association listservs, website listings of smoke-free housing and snowball sampling. We attempted to interview individuals who were involved in the smoke-free policy implementation process. We conducted 21 interviews from January through August 2016 [one company had two representatives participate in the interview, for 22 individual participants] which was sufficient to achieve saturation. The interviews were 60–90 min in length, and all but seven interviews were conducted over the phone. Interviewees gave signed or verbal consent and received a $25 gift card for their participation. All interviews were audio-recorded and transcribed verbatim. The study protocol and consent procedures were reviewed and approved by the Emory University Institutional Review Board.

### Interview guide

The interview guide was organized by steps in the policymaking process [i.e., development, adoption, implementation, maintenance] [27]. Specific questions covered reasons to adopt a smoke-free policy, players involved in the process, how policy details were decided, whether and how resident input was obtained, enforcement and compliance, and organization and community context (Additional file 1). Participants were asked to focus on the most comprehensive policy for which they were familiar with the policy-making and implementation process within their portfolio of properties.

### Data analysis

An initial codebook, based on the interview guide and a prior study of smoke-free policies in market rate housing, was refined after open coding of the first few transcripts by two coders. After coding several transcripts together to develop a common understanding of code definitions, the rest of the transcripts were double coded with discrepancies resolved through discussion. After all transcripts were double coded using NVivo 10, reports were generated for specific codes followed by a second round of inductive coding to identify themes within each code [28, 29]. We then created matrices to identify patterns by public versus affordable housing. Cells were populated with interview IDs to enhance trustworthiness of the findings with an audit trail [30].

## Results

### Study participants

Over half (59%) of those interviewed represented privately-owned affordable housing exclusively (Table 1). The rest managed a mix of market rate and affordable housing [41%]. Ten described properties in Georgia and 12 discussed properties in North Carolina. On average, the properties they described had 151 units each (ranging from 16 to 746). All had worked with the company for more than a year, with 36% having worked for the organization at least 10 years. The majority were women [59%], ages 36–50 [50%], and White [90%], with a college [59%] or graduate degree [23%]. At the time of the interview, 14% currently smoked.

**Table 1:** Description of Study Participants Representing Privately-Owned Affordable Housing

Characteristics	Representative^a^	
	N	Percent
State		
North Carolina	12	55
Georgia	10	45
Type^a^		
Privately-Owned Affordable Only	13	59
Mix of Market Rate and Subsidized Representatives	9	41
Tenure		
< 1	0	0
1–2	4	18
3–5	3	14
6–10	5	23
10+	8	36
unreported	2	9
Title		
CEO/President	2	9
Senior Vice President	4	18
Regional Manager	4	18
Regional Vice President	2	9
Property Manager	2	9
Other	8	36
Gender		
Male	9	41
Female	13	59
Age		
18–35	2	9
36–50	11	50
51–65	9	41
65+	0	0
Race/Ethnicity		
White	20	90
More than one race/Mixed	1	5
Other	1	5
Education		
High school graduate/GED	1	5
Some college/trade school/associates degree	3	14
College graduate	13	59
Post college graduate degree	5	23
Smoking Status		
Nonsmokers	15	68
Former smoker	4	18
Current smoker	3	14
E-Cigarette Use		
Never tried it	19	86
Tried it	3	14
Current user	0	0

^a^21 properties/management companies

### Description of the smoke-free policies

Table 2 describes smoke-free policies at the property discussed in the interview (some reported varying policies across properties but were asked to describe the most comprehensive policy). Four (19.0%) of the policies had been established prior to 2010, with 71% established between 2011 and 2014. Fewer than half (43%) banned smoking on the entire property. The rest (52%) banned smoking in all indoor areas or all common areas (5%). Three (14%) had designated outdoor smoking areas and eight (38%) had buffer zones of varying sizes. Almost half grandfathered smokers (i.e., allowed them to continue smoking in the unit), with 24% grandfathering for a limited time (usually 1 year) and 19% with unlimited grandfathering. Over one-third (43%) gave 6 months’ notice or less prior to policy implementation, and over half (52%) included e-cigarettes.

**Table 2:** Description of the Smoke-Free Policies at Referenced Properties ^a^

Policy Description	*N* = 21	%
Year of Policy Adoption		
Before 2010	4	19
2011–2014	15	71
2015 to present	2	10
Description of General Policy		
Comprehensive	9	43
All indoor spaces smoke-free	11	52
Common areas only	1	5
Description of Policies for Outdoor Spaces		
Buffer zones of 10, 15, 20 or 25 ft	8	38
No smoking anywhere except designated areas	3	14
Smoking allowed anywhere outside	0	0
Grandfathering of Smokers		
Yes – for a limited time	5	24
Yes – unlimited	4	19
Yes – length of time not specified	1	5
No	7	33
Not applicable (new build)	4	19
Notification Timeline		
No notification given	1	5
6 months or less	8	38
More than 6 months	7	33
Not applicable (new build)	2	10
Unclear	3	14
Inclusion of E-Cigarettes	11	52

^a^ Some participants reported varying policies across properties but were asked to describe policies at a property with the most comprehensive policy and with which they were most familiar

### Policy development

#### Impetus for the policy

Following a description of the smoke-free policy and affected properties, participants were asked to share how they or their company decided to adopt smoking restrictions at the reference property. Common reasons included: health, cost, fire, owner wishes, public norms and demand, HUD guidelines, new or rehabbed buildings, and organizational culture.

*We went to the meeting and I listened to this guy talking about it, and how it had improved their buildings, it had reduced their cost, they were replacing less carpet, they were paying less in painting, they were paying less on appliances that were smoked so bad they couldn't be used again, and I first – it first was a – truly a money saving thing to me. That was my first interest in it. And then the more we looked at it, it became – it was a good thing for health. It's just a good thing to support. And that's where it was born. –(Mixed, NC)*

#### Typical approval process

The typical approval process involved corporate leadership, Board members, owners and property managers. Residents were rarely involved in the decision-making process.

*Actually, it came from a corporate level. So Community Management kind of started the ball rolling, so to speak, and let the owners know that going smoke-free was kind of a thing to do now and was recommended. So the owners then had the final decision as to whether or not they wanted to do it or not. And they also were presented with options as to how to do it. Did they want to, you know, make the entire property smoke-free, did they want to grandfather certain people in, did they want to allow smoking in certain areas. So they kind of hashed through that and then told the management company what they wanted to do, and then it was up to us to implement it. (Affordable only, NC)*

When asked about resident input, about one-third explained there was no resident input, and another third discussed group meetings or educational sessions. Some described surveying residents and one-on-one meetings.

#### Deciding policy specifics

Drafting the policy required a series of decisions related to whether the policy should cover all outdoor and indoor areas, and if not, whether the policy should include designated smoking areas, buffer zones or both. Decisions about e-cigarettes became relevant more recently.

Reasons to go smoke-free property-wide included difficulty in identifying a good spot for a designated area, SHS infiltration from outside, smokers congregating at designated areas, costs associated with creating a designated area, and negative feedback from other managers about designated areas.

*I don't want to see a bunch of people smoking out the front door as we've got prospective guests or applicants coming in, and then if the residents open their windows, it may waft into there, and then – then if you start saying, well, we're going to designate an area – well, that's fine if you have a site that has that spot, but then you've also got to make it accessible, and then that costs money, to start pouring concrete, and then they say oh, inclement weather, and then you've got to put a roof – you know, it just kind of snowballs, so that's why we just said the heck with it ... (Affordable only, NC)*

Reasons not to go smoke-free property-wide included concern about alienating smokers, a belief that allowing smoking outdoors would encourage compliance with the indoor restrictions, and that it was easier to allow smoking on the property. Reasons for establishing a buffer zone and no designated smoking area included cost of the designated area, not wanting to create a gathering spot for smokers, and that government buildings have buffer zones. The perception that policies were easier to enforce was described as a reason for establishing a designated smoking area.

In the policies with e-cigarettes included (52%), the key reason was that it was easier to include everything (e.g., for enforcement reasons). Feeling unsure about the potential health and fire impacts was another reason to include them.

*Now, initially we had been allowing vapes because we thought that it was an opportunity for people to get off the cigarette. And for some it was. But we’re starting to see that there are logistical issues with that product, that option. Two logistical issues are that it does still leave smoke particles and it does still have a negative health impact, albeit lesser. But you can also put illegal drugs in the vape. (Mixed, GA)*

When policies did not include e-cigarettes, the most common reasons were that the policies were adopted before e-cigarettes became popular and that there was insufficient evidence about potential impacts.

#### Resources used in the policy development process

When asked to describe the resources used in developing their policies, participants mentioned attorneys who reviewed the new lease language, internet searches, health departments, HUD, other colleagues, and apartment associations.

*We received the smoke-free tool kit from HUD whenever they first came out with that and gave their examples of doing this in different locations [ … ] across the United States. So we decided that we would go ahead and try it. We followed their instructions, sent out a survey to the current tenants at the property that we wanted to implement this at, and just followed their basic outline, and then gave a certain amount of time once we received the surveys, decided where we would have the smoke-free area and the date that we would implement it. (Mixed, GA)*

### Implementation process

When asked to describe the implementation process and timeline, all participants described that the policy was included in a signed agreement--either a lease, lease addendum, or house rules. Residents were notified about the policy through lease signing and discussions. Giving 6 months’ advance notice was most common. Most participants said they offered residents cessation services, sometimes through a local health department but often through their resident services staff and other local partners. Signage was also mentioned as being key to implementation.

*We actually implement it as part of our house rules, and our house rules really are kind of what show all these items are what you have to follow, right? So the office hours are open from this to this, don't leave your trash outside, don't do this, don't do that. (Affordable only, GA)*

#### Enforcement of the smoke-free policy

Violations of the smoke-free policy were typically identified through routine unit maintenance inspections and resident reporting. This included, for example, adding smoking to existing inspection forms or the annual recertification unit inspection. All enforcement procedures included warnings, sometimes starting with verbal, but always including written warnings. The number of warnings varied, but warnings were typically followed by a lease violation notice, then a lease termination and/or eviction notice.

*When you do your property inspections, when you walk the units, that's when you need to find out and really start implementing this, and then you just treat it as any other lease violation, and to them that made sense, because they're so used to lease violations and following that guideline. (Affordable only, GA)*

Close to half described no enforcement challenges. Participants thought having a written policy, norms and culture of the housing community, public norms for smoke-free environments, and resident awareness of the rules and their consequences aided with compliance.

*I mean, it really has – it's become such a self-enforced policy now that parents don't want their kids walking through it, so they tell them to get out of the way and go stand out in the grass somewhere. I mean, it's – that's the part of it that I still, years later, have a hard time seeing, understanding, because I thought it would be very difficult to get to that point, and it's been very easy. (Affordable only, NC)*

When asked to describe challenges with enforcement, participants said that residents denied smoking or hid evidence, and that smell was often the only evidence. Participants also believed that concrete evidence, such as ashtrays or cigarette butts, would be required to prove residents were violating the policy. For evidence, some said that they took photos, kept written witness reports of violations, and/or kept air filters with nicotine residue. Another issue that complicated enforcement was the requirement to give 24-h’ notice before entering a unit, as did the lack of staff presence 24/7. Lastly, enforcement was viewed as more difficult on properties where buildings were spread out and did not share an interior hallway.

*One place we are having a difficult time is you have smokers. It’s on their clothes but you don’t find evidence of any ashtrays or anything so you go walk in the apartment, you still smell smoke. But it doesn’t seem strong enough that they are smoking in there and there are no ashtrays anywhere … . So that’s the one hard part that we have is determining whether they actually are smoking or they just go out and sit in their car and smoke and come back in and smell like it. (Affordable only, NC)*

Legal issues were also raised as an enforcement concern. Participants were worried that courts may not uphold an eviction, that smoke-free policies were not yet tested in their jurisdictions, or that they might need more concrete evidence. Residents going to legal aid or hiring lawyers and the costs of eviction court were also discussed.

*We have a case right now that – we have a disabled individual who we know is smoking in his apartment. We've cleaned – we've taken out two filters that are covered in nicotine. He smokes. And the moment we sent him a lease termination letter, he ran to legal aid. And of course legal aid is in the business to try to help people and they're trying to convince us to let him stay. They say, he's not smoking in the unit. Well, someone is smoking in the unit because we've got the filters to prove it. And he says, do you really want to go to court with a disabled person? And my answer was, I really don't care to go to court with a disabled person. The rules apply to the disabled person just like they do anyone else. No, we're not going to grant accommodations for someone smoking. First of all, I can't imagine that there's a doctor stupid enough to say that a person needs to smoke. But in the event that there was, no, we're not. That has been the biggest thing, is the tenant who gets a lawyer involved to try to keep you from putting them out, and then they claim they're not smoking. (Mixed, NC)*

### Actual lease termination/evictions

Over half of the participants had terminated leases or evicted residents due to smoke-free policy violations. Most of these were lease terminations, meaning they did not renew the resident’s lease at the time of annual renewal and did not go to court. Fewer than half of the properties had evicted one or more residents.

*...most of the people get the lease termination and they go, they really don't go to court. I don't know that we've taken but one to court, literally to court. The rest of them have just gotten the lease termination, they know they did wrong, and they left, so it's not really been too bad. (Mixed, NC)*

Most of those that had gone through the eviction process reported that the court upheld their order:

*She wouldn't quit and I went to court, and the judge said, is that your signature on the lease addendum, and she say, yes, and he said, judgment, he said, you signed it, you knew it, you're living here, and I'm sorry... (Mixed, NC)*

One participant reported that the court did not uphold an eviction:

*So we’ve had one person whose lease ended and was not invited to stay. Had one case where the person’s smoking turned into a fire. And they were let go. And we’ve had a recent case, so your call and our timing is timely, where we took it to court and the court decided that we could not evict. (Affordable only, NC)*

Overall, lease terminations and evictions were viewed as rare, with some participants stating their preference was to work with residents and not remove them from their housing:

*Sometimes a lease violation is ignored, okay? But when it gets to a lease termination, they realize it's serious and they realize that you are in fact enforcing the lease, and so sometimes they may come in and say, can I just give it one more shot, can I just become compliant, and we try to work with them, because you know, we don't want to put them out if we don't have to. The better option is to get them compliant, whether it's to totally quit smoking or at least cut back, but at least not to interfere with the rights of other residents in doing it. (Affordable only, NC)*

### Benefits of the smoke-free policy

Participants described a number of benefits accruing from the policy, with reduced turnover cost and time, and lower vacancy rates topping the list. Less litter, helping staff and residents to quit smoking, and reduced smell were also commonly mentioned. Health benefits, insurance credits or deductions, and fewer resident complaints were mentioned by a couple of participants, along with the policy bringing in a different type of resident/clientele, making the property nicer, less crime, fewer children smoking, happier residents, little impact on day to day work, fewer resident/neighbor issues, and fewer fires.

## Discussion

Understanding the smoke-free policy adoption, implementation, and enforcement process across a variety of housing contexts will help public health practitioners to share appropriate and applicable strategies, lessons learned, and best practices tailored to specific housing audiences. By comparing the process in privately-owned affordable housing to that of public housing and other housing types, we can identify useful similarities that may inform collaborative efforts to promote smoke-free housing across these different contexts. For example, reasons for adopting a smoke-free policy in the current study were essentially the same as reported in other studies of subsidized and market rate housing types, with health concerns, cost and fire topping the list [10]. However, more privately-owned affordable housing representatives in the current study mentioned reduced vacancy as a benefit than PHA representatives.

An important decision to make when developing a smoke-free policy in any setting is whether to establish a designated smoking area. Arguments against a designated area included no good location on the property, costs of creating them, and concern about smokers congregating. Similar arguments were presented against designated smoking areas by public housing representatives [25]. Those that chose to allow smoking on the property tended to believe it was less alienating to smokers and therefore could facilitate compliance. Similar views were shared in the ethnographic study by Anthony and colleagues [24].

Enforcement processes and challenges were also similar across public and affordable housing, with violations identified through routine inspections and resident reporting. Both used a series of warnings, followed by a lease violation notice, and then termination and/or eviction. Lack of staff presence in evenings and weekends was noted as an enforcement barrier in both types of subsidized housing. Our study also identified legal issues as an enforcement concern, with smoke-free policies not yet tested in their local jurisdiction, the perceived need for concrete evidence, and costs associated with eviction court described as additional challenges. These same concerns were expressed by PHA representatives [25, 26]. Lastly, challenges stemmed from resident denial and/or hiding of the evidence. Anthony et al. similarly reported strategies used by smokers to hide evidence of rule violations [24].

Some adoption, implementation, and enforcement processes do not appear to be universal across housing types. For example, in public housing, the typical approval process builds in some form of resident engagement, often through existing structures such as resident councils, resident representation on Boards, or required resident meetings [25]. However, in the current study, residents were rarely involved in the decision-making process, although a few mentioned surveying residents or holding meetings with residents after making the decision.

Other differences with public housing included shorter notification periods and more grandfathering of smokers in affordable housing. Grandfathering, not allowed in the new HUD rule, may reduce initial conflict with smokers, but causes other problems such as resentment and confusion for new residents who smoke and challenges with compliance [23, 25]. Privately-owned housing representatives did not discuss flexibility in the process as much as PHAs in Georgia and North Carolina, who more commonly described efforts to support smokers. PHA representatives were also more likely to collaborate with health departments for cessation services [25].

The findings should be considered in light of possible social desirability and recall bias. Participants knew the interviewers were affiliated with a school of public health and/or a state health department and were therefore supportive of smoke-free policies. We also interviewed just one representative per site; triangulating perspectives would have strengthened trustworthiness of the results. Strengths include the relatively large number of property management companies and clear saturation of themes.

## Conclusions

A deeper understanding of the smoke-free policy process in privately-owned affordable housing can help practitioners encourage policies in a range of subsidized housing contexts. The study identified salient benefits that can be highlighted when attempting to engage MUH partners, provides guidance on what to consider when designing smoke-free policies (e.g., layout, costs), and provides insights into enhancing compliance and managing enforcement. Future research could examine whether resident input and communication, methods for receiving complaints, and cessation assistance enhance compliance, and identify best practices for accelerating adoption in privately-owned affordable housing. Future research with rigorous measurement of policy implementation outcomes (e.g., compliance, reduced SHS exposure) could be leveraged to identify aspects of the policy implementation process that facilitate success. Understanding the type of evidence required by eviction courts would aid in the enforcement process as this was a common concern across public and privately-owned affordable housing. In the U.S., attention to government–subsidized housing not currently covered by the new HUD rule (e.g., housing choice vouchers, low income tax credit, tribal housing) is critical to reducing tobacco-related disparities in SHS exposure. Globally, understanding the range of MUH types and developing strategies to promote smoke-free policies in these settings is a high priority for reducing SHS exposure, especially among women and children.

## Additional file

Additional file 1: Privately Owned Affordable Housing PMO Interview Guide. Interview guide used with privately-owned affordable housing representatives. (DOCX 67 kb)

## Data Availability

The datasets generated and/or analysed during the current study are not publicly available due to their qualitative nature and difficulty in making them non-identifiable but are available from the corresponding author on reasonable request.

## Abbreviations

HUD: U.S. Department of Housing and Urban Development
MUH: Multi-unit Housing
PHA: Public Housing Authority
SHS: Secondhand Smoke
