# Supplementary material for: A qualitative study of the process of adoption, implementation and enforcement of smoke-free policies in privately-owned affordable housing
Source: BMC Public Health. 2019 Aug 8;19:1071. doi: 10.1186/s12889-019-7404-y (PMC6686249; doi:10.1186/s12889-019-7404-y)
Supplement: Supplementary file 1 — Privately Owned Affordable Housing PMO Interview Guide. Interview guide used with privately-owned affordable housing representatives. (DOCX 67 kb) [file 12889_2019_7404_MOESM1_ESM.docx]

**Investigating MUH smoke-free policy attitudes and implementation in North Carolina and Georgia**

**Guide 1: In-depth Interview with Property Managers/Owners in Privately-Owned Affordable Housing**

**Interview Overview**

**Length of Interviews:** 45-60 minutes

**Guide 1: Property Managers/Owners in Privately-Owned Affordable Housing**

**Aims:**

1. Identify similarities and differences in processes, facilitators and barriers to adoption, implementation, and enforcement of smoke-free policies or smoking restrictions in privately-owned affordable housing and public housing.
2. Examine equity issues, the role of residents and resident councils, and the awareness and perceived impact of HUD’s smoke-free rule.
3. In North Carolina, examine the awareness and perceived impact of North Carolina’s new tax credit.

**Sample: Affordable Housing Decision-makers who**

1. Operate privately-owned affordable (subsidized) multi-unit housing in North Carolina or Georgia, with some level of smoking restrictions.

**Interview Content**

1. Instructions for Interviewer
2. Introduction
3. Interview
   - Part 1: Background Information
     - Section A: General Property Characteristics
     - Section B: Existing Smoking-Related Policies
     - Section C: Property Details
     - Section D: HUD Rule
   - Part 2: Adoption, Implementation and Enforcement
     - Section E: Decision Making Process
     - Section F: Implementation & Enforcement Process
     - Section G: Impact of the Smoking Restrictions
   - Part 3: Context for Implementation
     - Section H: Organizational Context
     - Section I: Community Context
   - Part 4: Wrapping Up
     - Section J: Demographics
     - Section K: End the interview
4. Appendix

**Notes to interviewer**

1. Bolded and bracketed text contains instructions that should **not** be read aloud.
   1. For example: **“[If yes, Ask B3]”** tells you to proceed to Question B3 only if the respondent says yes to the current question.
2. Text in boxes provides instructions for how to proceed based on response.
3. A slash indicates text that you need to tailor. Do not read both words.
   1. For example: “Would/do you…”
4. Probes include information that we want to capture, but that the respondent may include in his/her answer to the primary question. Ask these follow-up questions only if the respondent does not address them. Related to probes, text in parentheses is additional information you can provide if the respondent is having trouble answering the question,
   1. For example: “Do you feel you have sufficient resources to implement the policy? (Resources could include material resources, financial resources, staff time, etc.)”
5. Text in italics is additional information for the interviewer (such as section headings) that is not to be read aloud.
6. It is possible that a respondent may address multiple questions when responding to one. Keep track of what has already been discussed and do not ask a question that was previously discussed.
7. Do not read answer options that are in all caps (e.g., REFUSED, DON’T KNOW)

**Interview Guide 1:**

**Property Managers/Owners in Privately-Owned Affordable Housing**

***Introduction***

Thank you so much for taking the time to speak/meet with me today. Before we get started I’m going to tell you a bit about the study and get your permission to continue.

**[Complete informed consent]**

Great, we are going to get started then. I am going to take notes as we are talking. But, as mentioned, I would also like to record our interview. That way I can go back after we finish and fill in anything I might have missed. May I have your permission to start recording?

***Part 1: Background Information***

***Section A: General Property Characteristics***

First I am going to ask for some background information about the properties that you own/manage and your roles.

A1. How many properties do you/does your company own or manage? **[If respondent manages a subset of the company’s properties, record both numbers]** __________________________

A2. What type(s) of property/ies do you own or manage? Would you call them a mix of market rate and federally subsidized housing, affordable housing, or some other type?

A3. What is your affiliation with this property/these properties? For example, are you the property owner, property manager, community manager, or something else?

A4. Could you tell me a bit more about your roles at the property/properties or your company?

- Probe: What do you do on a daily basis at work? What are your major responsibilities at the property/company?
- Probe: How many years have you worked on this property and/or for the company?

**[If answer to A1 = 1 property skip to Section B]**

A5. Where are these properties located?

Probe: In one region or across multiple regions?

A6. How many of your properties have some policy restricting smoking? ________________

**[If respondent unsure, ask for an approximate percentage]**

***Section B: Existing Smoking-Related Policies***

Now I’d like to ask about your property’s/ies’ current policies about smoking. By “policy”, I mean any of the rules, guidelines, or procedures that residents must follow about where they can or cannot smoke.

**[If owns/manages one property]:** You indicated that there is currently a policy in place prohibiting smoking at your property. **[Go to B1]**

**[If owns/manages multiple properties]**: You indicated before that your company currently has a policy in place prohibiting smoking in **[answer to A6]** of your properties.

Are there differences in policies at different properties?

**[If yes]:** How are they different?

In order to get more detail about how the policies are adopted and implemented, we would like to focus our discussion on one property. Do you have a property that was not a new build where smoking is restricted in some or all units?

**[If yes, go to B1]**

**[If no]:** Ok, then we would like you to think about the property with the most comprehensive smoking restrictions, including restrictions in some or all individual units. **[When participant has identified a property, go to B1]**

**[If no, go to B1]**

B1. Can you tell me in what areas is smoking prohibited according to policy? **[If owns/manages multiple properties with different policies]:** Please talk about the property that you just identified.

For example:

**[Note: only give option if it was not previously reported]**

- On the entire property?
- In common outdoor areas like entranceways, stairwells, breezeways or pool areas? Which ones?
- In common indoor areas, like indoor hallways, stairwells, the laundry room, etc.? Which ones?
- In private outdoor areas like patios and balconies? Which ones?
- In individual apartment units? **[If yes, ask B3]**
- In any other area?
- Is there a designated smoking area?

**[If smoking prohibited in apartment units]:**

B2. In how many apartment units is smoking prohibited? (If more than one property, refer to the previously decided upon property)

B3. Does this policy include a buffer zone, where smoking is prohibited within a certain number of feet (such as 25 feet) in any direction of the building?

B4. When were these restrictions first implemented?

**[If owns/manages multiple properties]**:

Is this same across all of your properties?

B5. What tobacco products are prohibited under your current policy? For example:

**[Only give option if it was not previously reported]**

- E-cigarettes (any electronic device that provides a vapor of liquid nicotine and/or other substances to the user as she or he simulates smoking)?
- Non-cigarette products (e.g. cigars, cigarillos, pipes)?
- Hookah?
- Non-combustible tobacco products (e.g. chewing tobacco, dip, snus, snuff)?

**MAKE NOTE OF RESPONSES TO B1-5 FOR FUTURE QUESTIONS**

***Section C: Property Details***

Now I’d like to get some specific details about this property we’ve been talking about.

**[If did not triage earlier (i.e., multiple properties with the same policy]** I’d like to get some specific details about how the smoke-free policy was adopted and implemented at one property. I’d like you to think of a property at which you have the most knowledge about the smoke-free policy. I’d like to get some specific details about that property.

C1. What is the total number of rental units (apartments) at this property?

C2. What building type is this property? For example, is it garden style, mid-rise, high-rise, townhomes, or some other type? *(Refer to Appendix for Building Type and Class definitions if needed)*

C3. What best describes the building class of this property? For example, are they Class A, B, C, D, or some other class type? *(Refer to Appendix for Building Type and Class definitions if needed)*

C4. Currently, how many rental units at this property are subsidized by U.S. Department of Housing and Urban Development (HUD) Low Income Public Housing funding (e.g., Section 8)?

**[If unsure]**

Would you say it is less than 25, 25-99, 100 or more?

C5. Do you have any units subsidized by other sources? What is/are the source/s?

C6. How is rent calculated for your subsidized units? For example, is it based on income, is it a percentage of the tenant’s income, or some other method?

C7. What is the average monthly rent a tenant pays for a one-bedroom and one-bathroom unit at your property? **[If property does not have one bedroom units, note monthly rent of the most common apartment size]**

Rent_____________________________ #Bed/Bath ___________________________

C8. Could you tell me more about the resident profile at this property?

**[If participant is unsure of the question]** What types of residents live at the property… e.g., young professionals? Families? Seniors? Students? Etc.

***Section D: HUD Rule***

Thank you for answering those background questions. Before we begin talking about the experience of putting smoking restrictions into place at your properties, I’d like to ask you a question about a new federal policy.

D1. HUD has proposed a rule that will prohibit smoking in all indoor spaces (including individual units) in public housing. What do you think about the proposed HUD rule?

**[If participant is not aware of the HUD proposed rule]:** The proposed rule mandates that lit tobacco be prohibited in all indoor spaces at public housing properties and PHA administrative offices. At this point, the rule does not cover subsidized units at private properties, such as Section 8 units. The proposed rule also mandates that smoking be prohibited in all areas within a 25 foot buffer zone around all buildings, including patios and balconies. There was a 60 day open comment period that closed January 19. Comments are being considered and a final rule will be published in the coming months. Once the rule is finalized, there will be an 18 month implementation period. What do you think about this rule?

D2. How do you think this rule will affect your properties?

***Part 2: Adoption, Implementation and Enforcement***

***Section E: Decision Making Process***

The following questions are specifically about the decision making process when you/your company first decided to adopt smoking restrictions on your property/ies. **[If owns/manages more than one property]** We are interested in the same property we were discussing in previous questions.

E1. Could you share how you/your company decided to adopt smoking restrictions at this property?

**[If respondent says that they were not involved in and/or have no knowledge of the decision-making process, skip to Section F]**

- - - Probe: What information or evidence influenced your decision? Where did you get this information?
    - Probe: Did the demographics of your residents impact your decision to adopt smoking restrictions? How do you see a smoking restrictions meeting the needs of your residents?

**[If owns/manages more than one property]**

- - - - Do you have other properties with smoking restrictions that have a different resident profile? How did that affect the adoption of your smoking restrictions?
    - Probe: You said earlier that your property is **[building type, answer to C2]**; did that impact your decision to adopt smoking restrictions?

**[If owns/manages more than one property]**

- - - Do you have other properties with smoking restrictions that are a different building type? How did that affect the adoption of your smoking restrictions?
    - Probe: You said earlier that your property is **[property type, answer to A2 ]**; did that impact your decision to adopt smoking restrictions?

**[If owns/manages more than one property]**

- - - Do you have other properties with smoking restrictions that are a different type of property? How did that affect the adoption of your smoking restrictions?

E2. What concerns did you/your company have about adopting smoking restrictions?

- - - Probe: Is there any particular type of resident you were most concerned about?

E3. Who was involved in the decision to adopt smoking restrictions?

- - - Did someone in your organization push for the smoking restrictions? **[If yes:]**
      - Can you tell me more about that?
      - Probe: What was their position? How did they push for it?
    - Was anyone within the organization against the policy? If yes:
      - Can you tell me more about that?
      - Probe: What was their position? How was that overcome?
    - Were any compromises made during the process? **[If yes:]**
      - Can you tell me more about that?
    - Who had to approve the decision? E.g. Board, executives?
    - How were residents involved in your decision-making process?

E4. How did you decide what spaces/areas to include when restricting smoking? For example, common outdoor areas, common indoor areas, individual units or the entire property **[refer to question B1]**.

- For those areas you decided not to include **[refer to B1]**, what was the rationale for leaving them out of the policy?

E5. How did you decide what types of tobacco products to include in the smoking restrictions? For example, cigarettes, pipes, cigars, smokeless tobacco and e-cig **[refer to the B5]**.

- **[If e-cigs/other products not included]** For those products that you decided not to include **[refer to question B5]** what was the rationale for leaving them out of the policy?

E6. When creating the policy to restrict smoking at the property, what resources did you use?

- - For example, where did you go for help? Who did you talk to? Who helped draft the lease language? (e.g., your apartment association, lawyers, tobacco control organizations, public health/healthcare organizations, etc.)
  - Did you receive any help from local or state health departments? **[If yes:]** Can you tell me more about that?

E7. Beyond the resources you had, what other resources would have been useful?

# ***Section F: Implementation & Enforcement Process***

# The next set of questions asks about how you implement and enforce your current smoking restrictions. Again, we’d like you to think about the property that we have been discussing.

***Initial Implementation* [only ask this section if respondent was present during planning/implementation of policy]**

F1. After the decision to adopt smoking restrictions was made, what initial steps did you take to actually put the policy into action on your property?

F2. Did you attempt to get buy-in from your residents? If so, how did you do it?

- Probe: Did you survey residents to see if they supported smoking restrictions?
- Probe: Did you hold meetings to see if they supported smoking restrictions?

F3. How did you notify the residents about the policy?

- Probe: Did you provide them a timeline before putting the restrictions into place? (i.e. were residents notified of when the policy would come into effect? or how much notice were residents before the policy would go into effect?)
- Probe: Is the policy included in their lease or some other written document?

**[If yes to lease/formal written document]**

When did you add it to existing tenants’ leases/community rules?

F4. Did you implement a grandfather clause?

F5. Did you offer your residents support and/or information about quitting smoking? (This could be a referral to a cessation program or quitline, holding classes on cessation, etc.)

**[If yes]:** Can you tell me more about the support and/or information you provided?

**[If no]:** Can you tell me more about that?

F6. What do you think about offering support and/or information on quitting smoking to residents who smoke? (This could be a referral to a cessation program or quitline, holding classes on cessation, etc.)

F7. How did the residents respond to the smoking restrictions?

- - Probe: Did different groups of residents react differently?
  - Probe: Who were the most vocal critics?
  - Probe: Who were the most supportive residents?
  - Probe: Did long-time residents respond differently than new residents?

F8. What processes do you have in place to get feedback from residents?

**[F9-F13 For upper management only]**

F9. How did you involve your staff in putting the policy in place?

- Probe: Was there any staff training regarding the policy?

**[If yes:]** What was that training like?

F10. Who is tasked with carrying out the implementation? i.e. who is in charge of putting the policy into place at the property level?

F11. How has the staff responded to the policy?

- - Probe: Have different staff responded differently? **[If yes]** Tell me more about that.

F12. What processes do you have in place to get feedback from staff?

***Overall Implementation Process* [ask of all participants]**

F13. Can you describe how the policy fits into your day to day operations? For example, how does it fit into your company’s existing practices, or your daily tasks?

- - - Probe: Has the policy changed the work you and your staff do on a day-to-day basis? Can you tell me more about this?
  - Probe: You said earlier that your property was a **[building type]**; can you tell me how that has impacted day to day implementation of the policy?
  - Probe: **[If participant responded earlier that there were different building types]:** How does day-to-day implementation differ in properties with other types of buildings?
  - Probe: You said earlier that your property was a **[property type – see A2]**; did that impact how you implemented your smoking restrictions?
    - Probe: Do you have other properties with smoking restrictions that are a different type of property? How does that affect the implementation of your smoking restrictions?

F14. Do you feel you had sufficient resources to implement the policy? (Resources could include material resources, financial resources, staff time, etc.) Tell me about that.

***Enforcement***

The following questions are about how you enforce your smoking restrictions. By enforce, I mean take action to see that your residents are actually following the policies.

F15. First, have you had any problems with people not following the policy? If so, please tell me about that.

F16. About how many residents have violated the smoking restrictions?

F17. What steps or procedures have you used to enforce the policy?

Probe: For example:

- Having a verbal discussion with residents?
- Giving written notices to residents after receiving complaints?
- Staff inspections?
- Fines? *(Note: move-out fees are different than fines. Clarify with respondent)*
- Terminating leases for residents who violate the policy?
- Evicting residents who violate the policy?

How effective were these methods?

F18. Are there any other enforcement methods in place that you have not had to use? **[If yes:]** Could you tell me more about that?

F19. How are your residents informed about the enforcement steps/processes associated with the smoking restrictions?

F20. How confident are you/your staff about enforcing the policy?

F21. What processes do you have in place for staff to report violations?

F22. What processes do you have in place for residents to report violations?

F23. What difficulties have you found in enforcing the smoking restrictions?

F24. What has helped with the enforcement process?

F25. What would have made the enforcement process easier?

# ***Section G: Impact of the Smoking Restrictions***

This next set of questions are about the impact of your current smoking restrictions.

G1. How do you think the policy is going?

- - Why do you say that?

G2. Could you tell us about some specific benefits you have noticed since putting the smoking restrictions into place?

Probe: Is there anything else you can think of? **[Do not provide choices]**

**[Only ask the following question if participants are upper management and did not have a comprehensive smoke-free policy (no smoking anywhere) in all properties or policy did not include E-cigs]**

G3. In the next 6 months, how likely is it that you/your company will **expand** your smoking restrictions to include other areas or tobacco products?

- Probe: Would you say very likely, somewhat likely, or not at all likely?

G4. Could you tell us more about why you think so?

**[If very likely or somewhat likely]:** How will you expand?

- - - Probe: What tobacco products/areas will be included?

**[If owns/manages properties with different levels of policy]** Have you considered expanding to your properties that don’t have a comprehensive smoke-free policy? Tell me more about that.

**[If not at all/somewhat likely]:** What might prevent you from expanding?

- - - Probe**:** Have you considered expanding the policy to other areas on your property?
    - Probe: Have you considered expanding the policy to include other tobacco products?

**[If owns/manages properties with different levels of policy]** Have you considered expanding to your properties that have fewer smoking restrictions? Tell me more about that.

**[For North Carolina PM/Os: ]**

G5. We understand that North Carolina is now (as of December 2015) requiring properties that receive the Low Income Housing Tax Credit to be smoke-free facilities. What influence, if any, did this policy change have on your decision to restrict smoking?

**[If owns/manages any properties without comprehensive smoke-free policies]**

Probe: How do you predict this policy change will impact the decision to restrict smoking at your properties in the future?

***Part 3: Context for Implementation***

***Section H: Organizational Context***

The next set of questions are about your organization.

***Culture***

H1. How do you think your management company's culture (general beliefs, values, assumptions that people embrace) has come into play with the implementation of the smoking restrictions?

H2. To what extent are new ideas embraced and used to make improvements in your company?

***Leadership and Relative Priorities***

H3. Could you describe the activities or initiatives that (appear to) have highest priority for your organization?

H4. **[For site managers]** What level of involvement has leadership at your organization had so far with the smoking restrictions?

- - Probe: Who are these leaders?
  - Probe: What kind of support have they given you? Can you provide specific examples?

# ***Section I: Community Context***

The last set of questions is about the housing industry and partnerships, as well as suggestions for promoting smoke-free policies or smoking restrictions in MUH.

***Cosmopolitanism***

I1. What kind of networking do you do with people from other management companies/other owners, either related to smoke-free policies, or more generally about your profession?

- - Probe: Are you a member of any professional organizations? Which ones? (e.g., an apartment association)
  - Probe: Listservs? Which ones?
  - Probe: Do you go to local or national conferences and/or Trainings? Which ones?

**[If yes:]** Have you heard about smoke-free policies or smoking restrictions from these sources? If yes, which ones?

Are there resources available for going smoke-free from any of these sources? Which ones?

I2. Where else have you heard about smoke-free policies or smoking restrictions and resources? (For example, HUD, health department, housing meetings and conferences)

I3. Who do you talk to if you have questions about smoke-free policies?

I4. What proportion of multi-unit properties (all properties, i.e. market, mixed, affordable and public) do you think are smoke-free in North Carolina/Georgia?

- - - Probe: How has this information influenced your decision to adopt a smoking restrictions?

***Interventions***

I5. Earlier, you mentioned that you received _______(assistance/resources)**[refer to E6]** from a health department when creating your policy.

[**If received help from a health department]**

Is there any specific assistance in addition to this that the Health Department could have provided? This could be during policy creation, implementation, enforcement or beyond.

**[If did not receive help from health department]**

Is there any specific assistance that the Health Department could have provided to help you with the process of restricting smoking on your property? This could be during policy creation, implementation, enforcement or beyond.

I6. We are considering developing a smoke-free certification process, in which property owners and managers could apply for their property to become certified as a smoke-free property.

What are your thoughts on a smoke-free certification process?

- - - Probe: Would you seek a smoke-free certification for your property/ies?
    - Probe: Would/Do you list “smoke-free” as an amenity? What do you think about that?

I7. If we were to develop a smoke-free certification what strategies could we use to promote it?

I8. What do you think would be essential to include in a smoke-free certification process? Anything we should not include?

I9. What other suggestions do you have for how public health professionals could accelerate adoption of smoke-free policies in affordable housing?

I10. We are almost done here, is there anything else you would like to share regarding smoke-free policies or restricting smoking in housing in general?

# ***Part 3: Wrap Up***

# ***Section I: Demographics***

Finally, I would like to ask you some general questions about yourself before we conclude the interview. These questions will help us better understand who is participating. As with the rest of the interview, you are welcome to skip any question, and your name and your property/company’s name will not be associated with your answers.

I1. What is your age? |______| years

I2. What is your gender? **[ASK ONLY WHEN IN DOUBT]**

MALE 1

FEMALE 2

REFUSED -7

DON’T KNOW -8

I3. Would you say that you are Hispanic or Latino?

YES 1

NO 2

REFUSED -7

DON’T KNOW -8

I4. What race and/or ethnicity do you consider yourself to be?

WHITE 1

AFRICAN AMERICAN OR BLACK 2

ASIAN/PACIFIC ISLANDER/NATIVE HAWAIIAN 3

AMERICAN INDIAN OR ALASKA NATIVE 4

MULTI RACIAL/MIXED 5

OTHER 6

REFUSED -7

I5. What is the highest level of school you completed or highest degree you received?

8TH GRADE OR LESS, 1

GRADES 9-12, 2

HIGH SCHOOL GRADUATE/GED, 3

SOME COLLEGE/TRADE SCHOOL/ASSOCIATES DEGREE,

4

COLLEGE GRADUATE, OR 5

POST GRADUATE DEGREE? 6

REFUSED -7

DON’T KNOW -8

I6. Have you smoked at least 100 cigarettes in your entire life?

YES 1

NO 2

REFUSED -7

DON’T KNOW -8

I7. During the past 30 days, on how many days did you smoke cigarettes?

I___I___I number of days

I8. Have you ever used an e-cigarette or other electronic “vaping” product, even just one time, in your entire life?

YES 1

NO 2

REFUSED -7

DON’T KNOW -8

**[If YES]**

I9. Do you now use e-cigarettes or other electronic “vaping” products every day, some days, or not at all?

EVERY DAY 1

SOME DAYS 2

NOT AT ALL 3

REFUSED -7

DON’T KNOW -8

***Section J: Ending the interview***

Lastly, we are hoping to interview 15-20 apartment residents in North Carolina and Georgia to better understand their thoughts about smoke-free policies in apartment buildings. Are there any residents from your property/ies with smoking restrictions who you think would be interested in speaking with us? They would also receive a $40 gift card for participating in a 45-60 minute interview.

- - **[If yes, collect contact info]** Thank you, please let them know that we will be in touch with them in the next week or so to schedule an interview.
  - **[If no]** Okay, please let us know if you do think of anyone. [provide contact information]

We are also still looking for other affordable housing properties and management companies with smoke-free policies. Do you know of any other companies with smoking restrictions who would be interested in speaking with us?

- - **[If yes, collect contact info]**
  - **[If no]** Okay, please let us know if you do think of anyone. [provide contact information]

Do you have an email address so that we can email you your gift card? [**If not, collect mailing address and ask for preference of Wal-Mart or Target]** You should receive it in the following week.

Email or physical address: _____________________________________

That concludes our interview today. Thank you so much for your time and input. Did you have any questions for me?

***APPENDIX***

**Building Type definitions from Fannie Mae:**

**Garden:** Multi-unit building in which each unit has its own entrance to the outside.

**Mid-rise:** Multi-unit with 3-5 floors.

**High-rise:** Multi-unit with 6 or more floors

**Townhouse:** 2 or 3-story unit with a common wall or walls bordering the adjacent unit. (Source: Redfin)

**Multifamily Property Classifications Overview**

**Class A Multifamily**

Generally, garden product built within the last 10 years

Properties with a physical age greater than 10 years but have been substantially renovated

High-rise product in select Central Business District may be over 20 years old

Commands rents within the range of Class “A” rents in the submarket

Well merchandised with landscaping, attractive rental office and/or club building

High-end exterior and interior amenities as dictated by other Class “A” products in the market

High quality construction with highest quality materials

**Class B Multifamily**

Generally, product built within the last 20 years

Exterior and interior amenity package is dated and less than what is offered by properties in the high end of the market

Good quality construction with little deferred maintenance

Commands rents within the range of Class “B” rents in the submarket

**Class C Multifamily**

Generally, product built within the last 30 years

Limited, dated exterior and interior amenity package

Improvements show some age and deferred maintenance

Commands rents below Class “B” rents in submarket

Majority of appliances are “original"

**Class D Multifamily**

Generally, product over 30 years old, worn properties, operationally more transient, situated in fringe or mediocre locations

Shorter remaining economic lives for the system components

No amenity package offered

Marginal construction quality and condition

Lower side of the market unit rent range, coupled with intensive use of the property (turnover and density of use) combine to constrain budget for operations

Source: http://www.crefcoa.com/property-classifications.html
